# The Integrin-Linked Kinase-PINCH-Parvin Complex Supports Integrin αIIbβ3 Activation

**DOI:** 10.1371/journal.pone.0085498

**Published:** 2013-12-23

**Authors:** Shigenori Honda, Hiroko Shirotani-Ikejima, Seiji Tadokoro, Yoshiaki Tomiyama, Toshiyuki Miyata

**Affiliations:** 1 Department of Molecular Pathogenesis, National Cerebral and Cardiovascular Center, Suita, Japan; 2 Department of Hematology and Oncology, Osaka University Graduate School of Medicine, Suita, Osaka, Japan; 3 Department of Blood Transfusion, Osaka University Hospital, Suita, Osaka, Japan; King's College London, United Kingdom

## Abstract

Integrin-linked kinase (ILK) is an important signaling regulator that assembles into the heteroternary complex with adaptor proteins PINCH and parvin (termed the IPP complex). We recently reported that ILK is important for integrin activation in a Chinese hamster ovary (CHO) cell system. We previously established parental CHO cells expressing a constitutively active chimeric integrin (αIIbα6Bβ3) and mutant CHO cells expressing inactive αIIbα6Bβ3 due to ILK deficiency. In this study, we further investigated the underlying mechanisms for ILK-dependent integrin activation. ILK-deficient mutant cells had trace levels of PINCH and α-parvin, and transfection of ILK cDNA into the mutant cells increased not only ILK but also PINCH and α-parvin, resulting in the restoration of αIIbα6Bβ3 activation. In the parental cells expressing active αIIbα6Bβ3, ILK, PINCH, and α-parvin were co-immunoprecipitated, indicating the formation of the IPP complex. Moreover, short interfering RNA (siRNA) experiments targeting PINCH-1 or both α- and β-parvin mRNA in the parent cells impaired the αIIbα6Bβ3 activation as well as the expression of the other components of the IPP complex. In addition, ILK mutants possessing defects in either PINCH or parvin binding failed to restore αIIbα6Bβ3 activation in the mutant cells. Kindlin-2 siRNA in the parental cells impaired αIIbα6Bβ3 activation without disturbing the expression of ILK. For CHO cells stably expressing wild-type αIIbβ3 that is an inactive form, overexpression of a talin head domain (THD) induced αIIbβ3 activation and the THD-induced αIIbβ3 activation was impaired by ILK siRNA through a significant reduction in the expression of the IPP complex. In contrast, overexpression of all IPP components in the αIIbβ3-expressing CHO cells further augmented THD-induced αIIbβ3 activation, whereas they did not induce αIIbβ3 activation without THD. These data suggest that the IPP complex rather than ILK plays an important role and supports integrin activation probably through stabilization of the active conformation.

## Introduction

 Cell adhesions are critical for hemostasis processes composed of interactions between vessel walls, platelets and coagulation-related proteins. During these processes, cells react with several elements such as extracellular matrix (ECM) proteins and cell surface receptors. As one of the main elements, an integrin family is known to play a key role in cell-ECM interactions. Integrins, transmembrane glycoprotein adhesion receptors, are composed of α and β subunits and are linked non-covalently. Both subunits include long extracellular domains, transmembrane domains, and short cytoplasmic domains. There are at least two conformational states of integrin presenting low affinity (inactive) or high affinity (active) against its ligands and this heterodimeric receptor acts as a bidirectional signaling transducer. The binding of the cytoplasmic proteins such as talin and kindlins to the integrin β cytoplasmic domain upregulates the ligand-binding affinity of integrin (inside-out signaling). In contrast, ligand binding to integrins and the subsequent clustering of ligand-bound integrins result in intracellular molecular rearrangements such as focal adhesion formation and cell spreading (outside-in signaling) [1].

αIIbβ3, a major integrin expressed on platelets, is critical for platelet aggregation mediated by bindings of fibrinogen and von Willebrand factor. Since inside-out signaling pathways of αIIbβ3 induce striking conformational changes between inactive and active states, the activation processes of αIIbβ3 have been extensively investigated [2]. Talin, a cytoskeletal protein consisting of an N-terminal head and a C-terminal rod, has been well characterized as an integrin activator [3,4]. The talin head domain (THD) contains four subdomains: F0, F1, F2, and F3. The F3 domain itself can bind to the β3 cytoplasmic domain and exert αIIbβ3 activation [5]. Other subdomains also have important roles in the activation [6-8]. The kindlin family members (kindlin-1, -2, and -3), which are focal adhesion proteins, have recently been proven to be critical for integrin activation [9,10]. Kindlin-1 and -2 are widely expressed and kindlin-3 expression is restricted mainly to hematopoietic cells [11]. Several studies suggest that the binding of talin and kindlins to the integrin β3 cytoplasmic domain is pivotal for the final step in the inside-out activation of αIIbβ3. Moreover, since kindlins synergistically augment talin-dependent αIIbβ3 activation, they act as a co-activator of talin [12,13]. However, regulatory molecules other than talin and kindlins necessary to αIIbβ3 activation remain to be fully clarified.

 Since platelets are inadequate for gene manipulation, the CHO cell system has been used to study essential regulators of integrin αIIbβ3 function. For example, αIIbβ3-expressing CHO cells contributed to the elucidation of the functional importance of kindlin-1 and -2 as co-activators and of THD as a direct activator of integrin [10,12]. It was also shown that the Rap1-GTP-interacting adaptor molecule promotes talin-dependent integrin activation in the CHO cell system [14]. A chimeric integrin, αIIbα6Aβ3β1 or αIIbα5β3, expressed on CHO cells having the extracellular and transmembrane domains of αIIbβ3 connected to the cytoplasmic domains of α6Aβ1or α5β3 has been constitutively active on CHO cells but susceptible to integrin regulatory proteins [15]. Several integrin regulatory proteins including H-ras, PEA-15, CD98, and talin were characterized in this cell system [15-18]. Thus, the CHO cell system has been utilized to analyze the mechanisms by which integrin function is regulated. 

 Integrin-linked kinase (ILK) was originally identified as a serine/threonine kinase associated with integrin β1 and β3 cytoplasmic domains. It consists of three domains: an N-terminal ankyrin repeat domain, a putative pleckstrin homology domain, and a C-terminal kinase domain [19]. Many studies have shown that ILK is widely expressed and involved in interactions between integrins, cytoskeletal proteins, and signaling molecules. A deficiency or aberrant function of ILK resulted in the impairment of adhesion, spreading, migration, proliferation, and survival of the cells [20]. ILK seems to have two functions: that of a scaffold protein and that of a protein kinase, whereas the kinase activity is controversial [21,22]. At focal adhesion sites, ILK forms a heterotrimeric complex composed of the adaptor proteins PINCH and parvin [23-28]. PINCH consists of two members, PINCH-1 and -2, each of which consists of five LIM domains. PINCH-1 and -2 are ubiquitously expressed in mammalian tissues and show overlapping expression in many tissues. Parvin comprises three members, α-, β-, and γ-parvin, and contains N-terminal nuclear localization sequences and two calponin homology domains. In mammalian tissues, α- and β-parvin are ubiquitously expressed but γ-parvin is expressed mainly in hematopoietic tissues. These adaptor proteins are known to directly bind to several cytoplasmic proteins including Nck2 for PINCH and filamentous actin for parvin [25,29]. The ankyrin repeat domain of ILK binds to PINCH and the kinase domain binds to parvin. ILK interacts directly or indirectly with several other cytoskeletal and signaling proteins and exerts diverse roles in different tissues [30].

 In our previous study, we identified ILK as a molecule important for integrin activation, using an expression cloning system as follows. First, we established CHO cells expressing constitutively active integrin αIIbα6Bβ3 whose αIIb cytoplasmic domain we replaced by that of integrin α6B (parental cells). Next we obtained mutant cells with inactive integrin using genome-wide mutagenesis, and finally isolated an ILK cDNA was isolated as a factor that complements the function of inactive αIIbα6Bβ3 in mutant cells by expression cloning [31]. Although the role of ILK at focal adhesion sites has been well studied, there are only a few reports on the involvement of ILK in integrin activation [32,33]. In the present study, we further investigated the mechanisms by which ILK regulates integrin activation in the CHO cell system. 

## Materials and Methods

### Plasmids

 Human wild-type (WT) αIIb and β3 subcloned into expression plasmid pcDNA3 (Invitrogen, San Diego, CA) were provided by Drs P. Newman and G. White (Blood Research Institute, Blood Center of Wisconsin, Milwaukee, WI). pRKHA, including full-length mouse talin-1 was a gift from Dr K. Yamada (NIH, Bethesda, MD). The N-terminal head region (residues 1-433) of talin-1 was constructed by polymerase chain reaction (PCR) and subcloned into green fluorescence protein (GFP) containing vector pEGFP-N1 to make a fusion protein of THD with GFP (THD-GFP) (Clontech, Mountain View, CA). Mouse α-parvin cDNA and mouse PINCH-1 cDNA (Thermo Scientific Open Biosystems, Lafayette, CO) were amplified by PCR and then were subcloned into expression plasmids, pcDNA3.1 (Invitrogen) for α-parvin and pBApo-CMV Pur DNA (Takara Bio, Shiga, Japan) for PINCH-1. pcDNA3-αIIbα6B was created using PCR-based mutagenesis as previously described [31]. Nucleotide and amino acid numbers begin with the start codon (ATG) and the first Met residue, respectively. The full length of rat ILK cDNA was amplified by PCR then subcloned into pcDNA3 and GFP-encoding plasmid pAcGFP1-Hyg-C1 to make a fusion protein of ILK with GFP (GFPILK-WT) (Clontech). Three point mutations (H99D/F109A/W110A) in the ankyrin repeat domain of ILK were introduced into pAcGFP1-Hyg-C1 to make a fusion protein of the ILK mutant with GFP (GFPILK-H99D/F109A/W110A). Two point mutations (M402A/K403A) in the ILK kinase domain were introduced in pAcGFP1-Hyg-C1 to make a fusion protein of the ILK mutant with GFP (GFPILK-M402/K403A). The ILK mutant (H99D/F109A/W110A) was designed to disrupt the PINCH binding based on the crystal structure of a complex of the ankyrin repeat domain of ILK with the LIM1 domain of PINCH, PDB 3F6Q [34]. The ILK mutant (M402A/K403A) was designed to disrupt the parvin binding as previously reported [35]. Expression plasmid pCMV-SPORT6, containing full-length mouse kindlin-2 (Thermo Scientific Open Biosystems) was obtained. All PCR-generated DNA inserts were verified by sequencing using a BigDye Terminator Cycle Sequencing Kit (Applied Biosystems, Foster City, CA).

### Cell Cultures

 CHO-K1 cells from ATCC were cultured in DMEM supplemented with 10% fetal bovine serum and 1% non-essential amino acids (Sigma-Aldrich, St. Louis, MO). CHO cells stably expressing constitutively active αIIbα6Bβ3 (parental cells) were previously established [31]. CHO-K1 cells were cotransfected with pcDNA3-αIIbα6B and pcDNA3-β3 using Lipofectamine 2000 (Invitrogen) and selected with 1 mg/ml G418 (Nacalai Tesque, Kyoto, Japan). G418-resistant cells expressing αIIbα6Bβ3 were cloned to isolate parental cells by a limiting dilution method. ILK-deficient mutant cells, which result in its inactive form from active αIIbα6Bβ3 (mutant cells), were previously established by the introduction of random mutations into the parental cells using a chemical mutagen, ethyl methane sulfonate (EMS) [31]. For αIIbβ3-expressing CHO cells, pBApo-CMV Pur DNA-αIIb and pcDNA3-β3 were cotransfected into CHO-K1 cells by Lipofectamine 2000. After selection with 12 μg/ml puromycin (Clontech) and 1 mg/ml G418, clones expressing αIIbβ3 were established by the limiting dilution method. 

### Flow cytometry

 Flow cytometry analyses were performed as previously described [31]. Cells suspended in Tyrode’s buffer containing 1.5 mM CaCl_2_, 1 mM MgCl_2_, and 1% bovine serum albumin were incubated with the primary antibody of 5 μg/ml of a mouse monoclonal antibody (mAb) specific for αIIbβ3, HIP8 (BD Biosciences) for 30 minutes at 4°C. After washing, the cells were incubated with the secondary Ab of ~1 μg/ml phycoerythrin (PE)-conjugated rat anti-mouse IgG (BD Biosciences) for 30 minutes at 4°C, washed once, stained with 1 μg/ml 7-aminoactinomycin D (7AAD) (Sigma-Aldrich) to discriminate dead cells, and then analyzed on a flow cytometer (FACSCalibur; BD Biosciences). As a negative control, cells were incubated with the secondary Ab alone. For the binding of a ligand-mimetic, activation-specific anti-αIIbβ3 mAb, PAC-1 (BD Biosciences), cells were incubated with 10 μg/ml PAC-1 for 30 minutes at room temperature in the absence or presence of 10 μM of a peptidomimetic antagonist of αIIbβ3, Ro44-9883 (a gift from Astellas Pharma, Tokyo, Japan), washed once, and then incubated with 10 μg/ml PE-conjugated anti-mouse IgM (eBioscience, San Diego, CA) for 30 minutes at 4°C. After washing, cells were stained with 7AAD and then analyzed. As a positive control for PAC-1 binding, cells were incubated with 15 mM dithiothreitol (DTT) for 10 minutes at 37°C to activate integrin αIIbα6Bβ3 and incubated with PAC-1 as mentioned above. Integrin activation was quantified as an activation index calculated using the following formula: 100 x (*F* − *Fo*) / (*F*max − *Fo*), where *F* is the median fluorescence intensity (MFI) of PAC-1 binding, *Fo* is the PAC-1 binding in the presence of Ro44-9883, and *F*max is the maximal PAC-1 binding in the cells treated with DTT. For fibrinogen binding, cells were incubated with 150 μg/ml Alexa-Fluor 647-conjugated fibrinogen (Molecular Probes, Eugene, OR) under similar conditions to the above assay. In some experiments using αIIbβ3-expressing CHO cells, the activation indexes were normalized by αIIbβ3 expression, as shown by the following formula: 100 x (*F* − *Fo*) / (F_*1*_ - F_*2*_), where *F* and *Fo* are the same as mentioned above, *F*_*1*_ is the HIP8 binding, and *F*_*2*_ is the binding of the secondary Ab alone. 

### Immunoblotting

 Immunoblotting was performed using procedures previously described [31]. In brief, cell lysates were subjected to sodium dodecyl sulfate-polyacrylamide gel electrophoresis (SDS-PAGE) and transferred to a polyvinylidene difluoride (PVDF) membrane. The membranes were incubated with either one of the following primary Abs: 0.125 μg/ml mouse mAb specific for ILK, 3/ILK (BD Biosciences), 0.25 μg/ml mouse mAb specific for PINCH, 49/PINCH (BD Biosciences), rabbit polyclonal Ab specific for α-parvin (IgG fraction; 1:3,000) (Sigma-Aldrich), 1 μg/ml mouse mAb specific for β-parvin, 11A5 (Millipore, Temecula, CA), mouse mAb specific for talin, 8D4 (ascites fluid; 1:2,000) (Sigma-Aldrich), rabbit polyclonal Ab specific for kindlin-2 (IgG fraction; 1:1,000) (ProteinTech Group, Chicago, IL), 0.5 μg/ml rabbit polyclonal Ab specific for glyceraldehyde-3-phosphate dehydrogenase (GAPDH), FL-335 (Santa Cruz Biotechnology), horseradish peroxidase (HRP)-conjugated rabbit polyclonal Ab specific for β-actin (IgG fraction; 1:2000) (MBL, Woburn, MA), or 1 μg/ml mouse mAb specific for GFP, B-2 (Santa Cruz Biotechnology) for 90 minutes at room temperature. After washing, bound Abs except for the HRP-conjugated Abs were incubated with peroxidase-conjugated secondary Abs (Kirkegaard & Perry Labs, Gaithersburg, MD) Detection was performed using a chemiluminescence kit (Immobilon Western; Millipore, Bedford, MA). Chemiluminescence was visualized by an image analyzer, LAS-3000PLUS (Fuji Photo Film, Kanagawa, Japan).

### Immunoprecipitation

 Parental cells were solubilized at concentrations of 2 x 10^7^ cells/ml in a lysis buffer (150 mM NaCl, 50 mM Tris-HCl, pH 7.5, and 1% Triton X-100) containing proteinase inhibitors. After centrifugation at 15,000 x g for 12 min, the supernatant (200 μl) was subjected to immunoprecipitation using protein A/G agarose (Santa Cruz Biotechnology) and the following Abs: 1 μg mouse mAb specific for ILK, 3/ILK, 1 μg mouse mAb specific for PINCH, 49/PINCH, 1 μg mouse IgG_1_ isotype control, MOPC 21 (Sigma-Aldrich), 1 μg mouse IgG_2a_ isotype control, UPC-10 (Sigma-Aldrich), 1 μg rabbit polyclonal Ab specific for α-parvin, and 1 μg pooled rabbit IgG (Invitrogen). The immunoprecipitants were analyzed by immunoblotting as described above. As a positive control, cell lysates (15 μl) were loaded onto a lane. 

### Short interfering RNA (siRNA) and transfection

 Total RNA from parental cells was extracted with Trizol reagent (Invitrogen). PINCH-1, α- and β-parvin, and kindlin-2 mRNA were amplified by a one-step RT-PCR kit (Qiagen, Valencia, CA) using primers specific to both mouse and rat homologues according to the manufacturer’s instructions. RT-PCR products were directly sequenced using specific primers. 

 siRNAs against RNA targets were designed and synthesized by Invitrogen (Stealth RNAi). The siRNA target sequences of hamster mRNA are as follows: PINCH-1 (p) 157 sense 5’-CGGGUUAUUAAAGCCAUGAACAACA-3’; PINCH-1 (p) 755 sense 5’-CCTGCAATACCAAATTAACACTCAA-3’; α-pavin (pa) 503 sense 5’-CCAGGAGCATCAAGTGGAATGTAGA-3’; α-pavin (pa) 761 sense 5’-CAGACAAGCTCAACGTGGTAAAGAA-3’; β-pavin (pb) 900 sense 5’-UCCACAACUUCUACCUGACACCUGA-3’; β-pavin (pb) 1011 sense 5’-AAGAUGUGGUAAACUUGGACCUCAA-3’; kindlin-2 (k) 770 sense 5’-GAUCGCUAAUGGAACAAGAUGUGUGAA-3’; kindlin-2 (k) 770 scrambled control sense 5’-GAUAUCGUAAAGAACUAGUGCGGAA-3’; kindlin-2 (k) 1733 sense 5’-AAGGCGGCAAGAGAGAAGAACUUAU-3’; kindlin-2 (k) 1733 scrambled control sense 5’-AAGCGGGAAAGAAAGAAGUCGCUAU-3’. The sequences of ILK siRNA (Ilk1255) and its scrambled control were previously described [31]. Stealth RNAi-negative control duplexes (Invitrogen) were used as controls in knockdown experiments targeting PINCH-1 and parvins. Cells cultured in six-well plates were transfected with 12.5-50 nM siRNA using Lipofectamine RNAiMAX (Invitrogen) or cotransfected with 30 nM siRNA and the indicated amounts of plasmid (0.5 μg pEGFP-N1 encoding THD-fused GFP or 0.015 μg pEGFP-N1 plus 0.485 μg pcDNA3 as a negative control) using Lipofectamine 2000. Transfected cells were usually analyzed at 72 hours after transfection. For transfections with plasmid DNA alone, Lipofectamine 2000 was employed.

### Statistical analysis

 The statistical significance of observed data was determined using one-way analysis of variance followed by Bonferroni’s post hoc test using PRISM 5 software (GraphPad Software; La Jolla, CA, USA). P values of 0.05 or less were considered statistically significant. 

## Results

### Evaluation of PINCH, α-parvin, talin, and kindlin-2 in ILK-deficient αIIbα6Bβ3- inactive mutant CHO cells and αIIbα6Bβ3-active parental CHO cells

**Figure 1:**
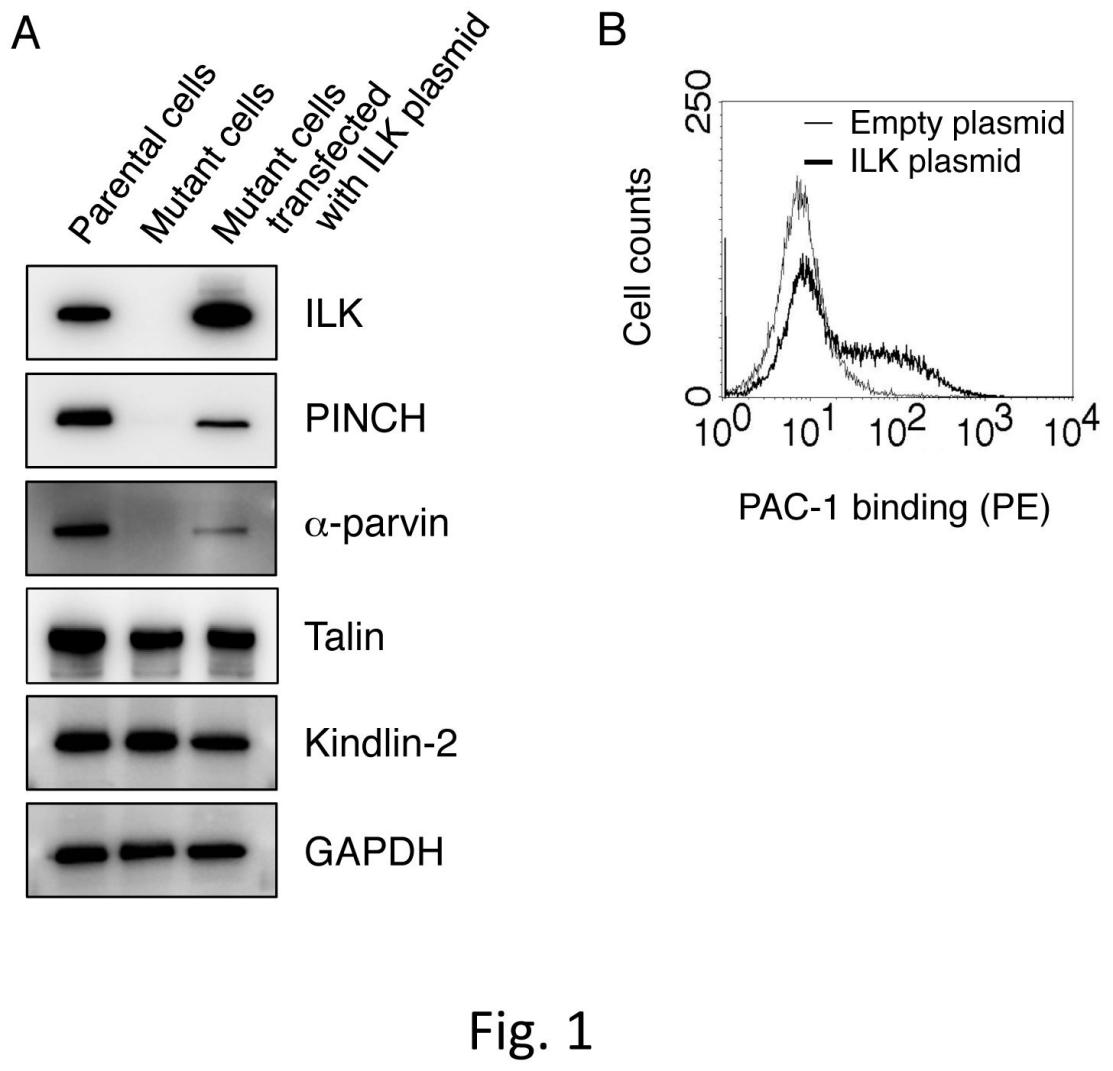
Characterization of ILK-deficient mutant cells expressing inactive αIIbα6Bβ3. (A) Immunoblotting for ILK, PINCH, α-parvin, talin, and kindlin-2. Cell lysates obtained from parental cells with constitutively active αIIbα6Bβ3, ILK-deficient mutant cells with inactive αIIbα6Bβ3, and mutant cells transiently transfected with rat ILK cDNA were electrophoresed on SDS-PAGE gels and immunoblotted with indicated Abs. GAPDH shows an internal loading control. (B) Flow cytometry analysis showing PAC-1 (an activation-specific mAb for αIIbβ3) binding to mutant cells transiently transfected with either ILK plasmid or empty plasmid. Bound PAC-1 was detected with a PE-conjugated secondary mAb.

 We previously obtained ILK-deficient mutant cells by treating parental cells expressing constitutively active αIIbα6Bβ3 with the chemical mutagen EMS [31]. In the mutant cells, ILK mRNAs contained two nonsense mutations, R317X and W383X, in a compound heterozygous state, resulting in a complete lack of ILK expression. It has been shown that ILK forms a ternary complex with PINCH and parvins to make an IPP complex [25]. To assess the role of ILK-binding proteins in ILK-deficient mutant cells with the inactive state of αIIbα6Bβ3, we examined the protein expression of ILK-binding adaptor proteins, PINCH and α-parvin. In addition to a lack of ILK expression, mutant cells showed severe reductions in the protein expression of PINCH and α-parvin as compared to parental cells. In contrast, talin and kindlin-2, which play critical roles in integrin activation, were present at normal levels of protein expression (Figure 1A). Transfection of a plasmid encoding ILK cDNA into mutant cells showed the increased expression of ILK and concomitant increases in PINCH and α-parvin expression levels but did not affect talin and kindlin-2 expression levels. Moreover, flow cytometry using an activation-specific anti-αIIbβ3 mAb, PAC-1, showed that ILK-plasmid transfection increased PAC-1 binding compared to empty-plasmid transfection (Figure 1B). These data suggest that ILK, PINCH, and α-parvin are necessary to restore the active state of αIIbα6Bβ3 in mutant cells. 

### Detection and assessment of ILK, PINCH, and parvin (IPP) complex in αIIbα6Bβ3-active parental cells

**Figure 2:**
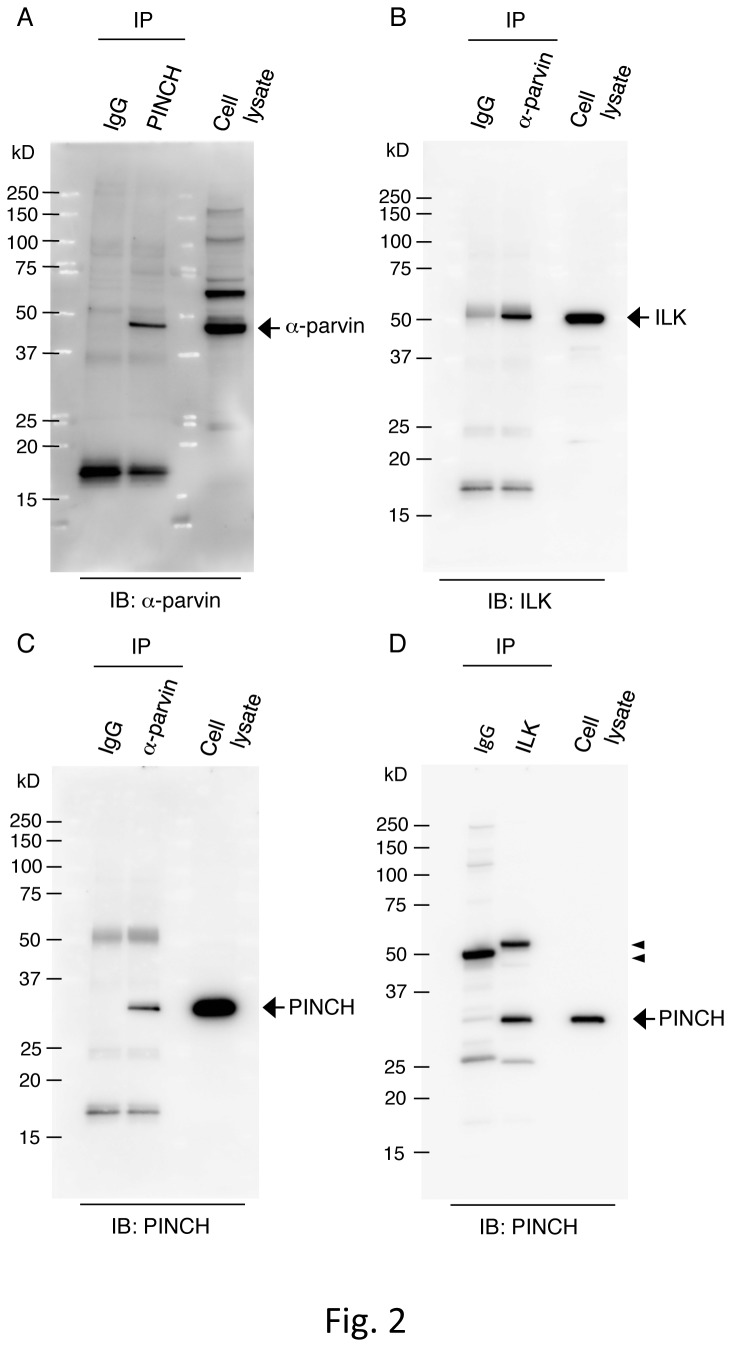
Detection of IPP complex proteins in αIIbα6Bβ3-active parental cells. Cell lysates obtained from αIIbα6Bβ3-active parental cells were immunoprecipitated with Abs against PINCH (A), α-parvin (B, C), and ILK (D). The co-precipitates were detected by Abs for α-parvin (A), ILK (B), and PINCH (C, D). IgG means immunoprecipitation (IP) using non-immune control IgG. IB stands for immunoblotting. Arrows indicate the predicted sizes of the indicated proteins. Arrowheads (D) indicate the antibody heavy chains used in the IP. Different mobilities between those of the two IgG antibodies are probably caused by differences in the amino acid compositions of them.

**Figure 3:**
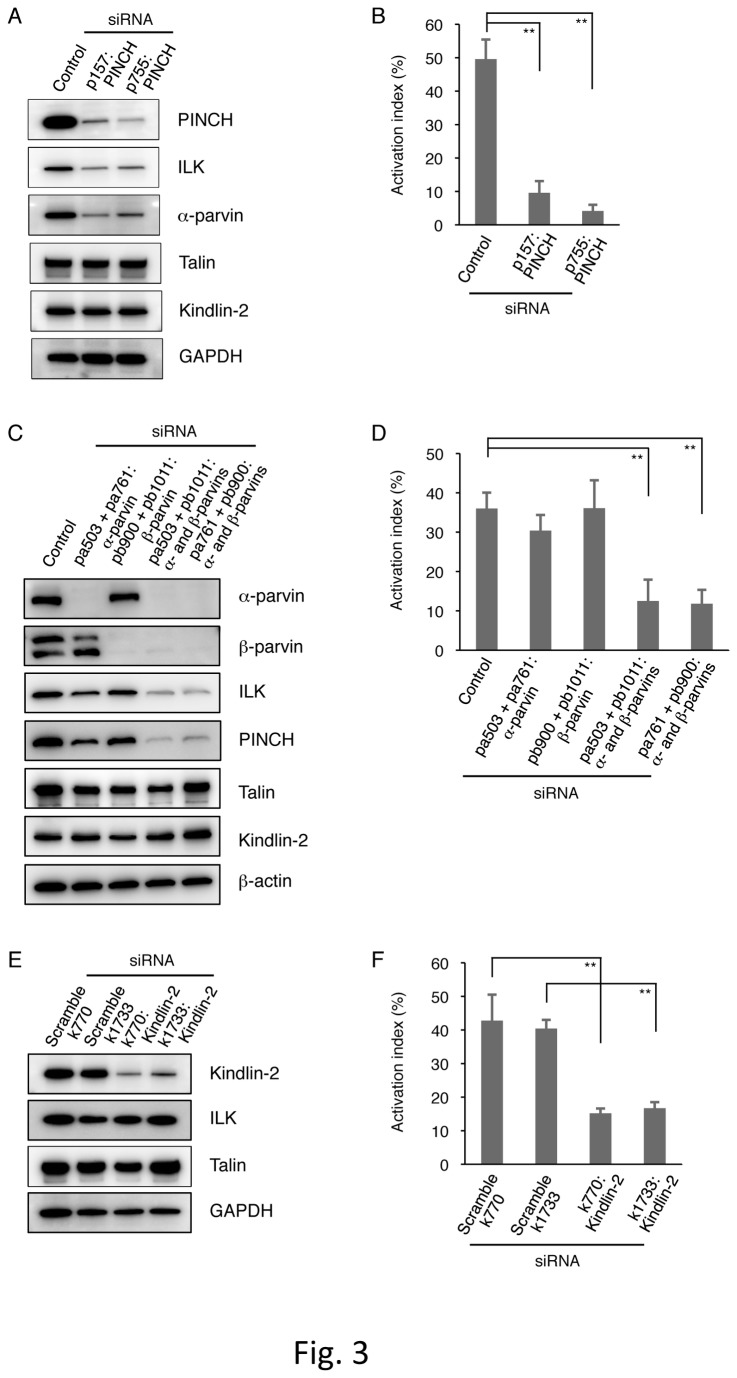
Knockdown effects of PINCH, parvins, and kindlin-2 in αIIbα6Bβ3-active parental cells. αIIbα6Bβ3-active parental cells were transiently transfected with PINCH siRNAs (p157 and p755) (A), α-parvin siRNAs (pa503 and pa761) (C), β-parvin siRNAs (pb900 and pb1011) (C), kindlin-2 siRNAs (k770 and k1733) (E), negative control siRNAs, and scrambled siRNAs. Cell lysates were electrophoresed on SDS-PAGE gels, and the separated proteins were immunoblotted with the indicated Abs. GAPDH and β-actin are shown as internal loading controls. The activation indexes of transfected cells (B, D, F) were calculated using the formula shown in Materials and Methods. A value of 100% implies the maximum PAC-1 binding to the cells treated with dithiothreitol (DTT). Data represent means ± standard deviation (SD) of three (B, F) or four (D) independent experiments. ** indicates *P* < 0.01.

 Since ILK, PINCH, and parvins form the IPP complex, we assessed IPP complex formation in αIIbα6Bβ3-active parental cells, which show constitutively active αIIbα6Bβ3. Immunoprecipitation experiments revealed that ILK is coprecipitated with PINCH and α-parvin, indicating the presence of the IPP complex in those cells (Figure 2). To evaluate the importance of these proteins comprising the complex on the active state of αIIbα6Bβ3, we performed RNA interference (RNAi) experiments targeting PINCH or parvins, and we analyzed the active state of αIIbα6Bβ3 by flow cytometry using PAC-1. For PINCH siRNA, we targeted PINCH-1, one of the two PINCH isoforms, because we failed to find sequences of PINCH-2 mRNA in CHO cells. Each of the two PINCH-1 siRNAs (p157 and p755) decreased PINCH expression and concomitantly decreased ILK and α-parvin expression compared to nontargeting negative control siRNA in parental cells (Figure 3A), leading to a decreased integrin activation index, which was determined by flow cytometry analysis of PAC-1 binding (Figure 3B). For parvin siRNA, α- and β-parvins were knocked down since both parvins are thought to bind to ILK. A mixture of two α-parvin siRNAs (pa503 and pa761) or two β-parvin siRNAs (pb900 and pb1011) reduced α-parvin or β-parvin expression, respectively; however, ILK and PINCH expression levels were less significantly affected (Figure 3C). When a mixture of α-parvin and β-parvin siRNAs was transfected into parental cells, the expression levels of α-parvin and β-parvin were decreased and concomitant decreases in ILK and PINCH expression were observed. Flow cytometry analysis evaluating the activation state exhibited that the transfection of both α- and β-parvin siRNAs, but not that of either α- or β-parvin siRNA significantly decreased PAC-1 binding (Figure 3D). These data suggest that the IPP complex formation of ILK, PINCH, and parvins is necessary for αIIbα6Bβ3 activation in a CHO cell system. 

### Knockdown of kindlin-2 in αIIbα6Bβ3-active parental cells

 In our previous work, talin siRNA decreased PAC-1 binding to αIIbα6Bβ3-active parental cells [31]. To confirm that kindlin-2 plays an important role in integrin activation in the CHO cell system, we performed the kindlin-2 siRNA experiment in parental cells (Figure 3E, F). Each of two different siRNAs (k770 and k1733) reduced kindlin-2 expression and decreased PAC-1 binding in association without significantly affecting ILK or talin expression. In addition, when an ILK cDNA was cotransfected with kindlin-2 siRNA into ILK-deficient mutant cells, PAC-1 binding was decreased as compared to those of the cotransfection of both the ILK cDNA and scrambled kindlin-2 siRNA (data not shown). These data indicate that kindlin-2 is required for αIIbα6Bβ3 activation in parental cells. 

### Role in binding of ILK to PINCH and parvin for integrin activation

**Figure 4:**
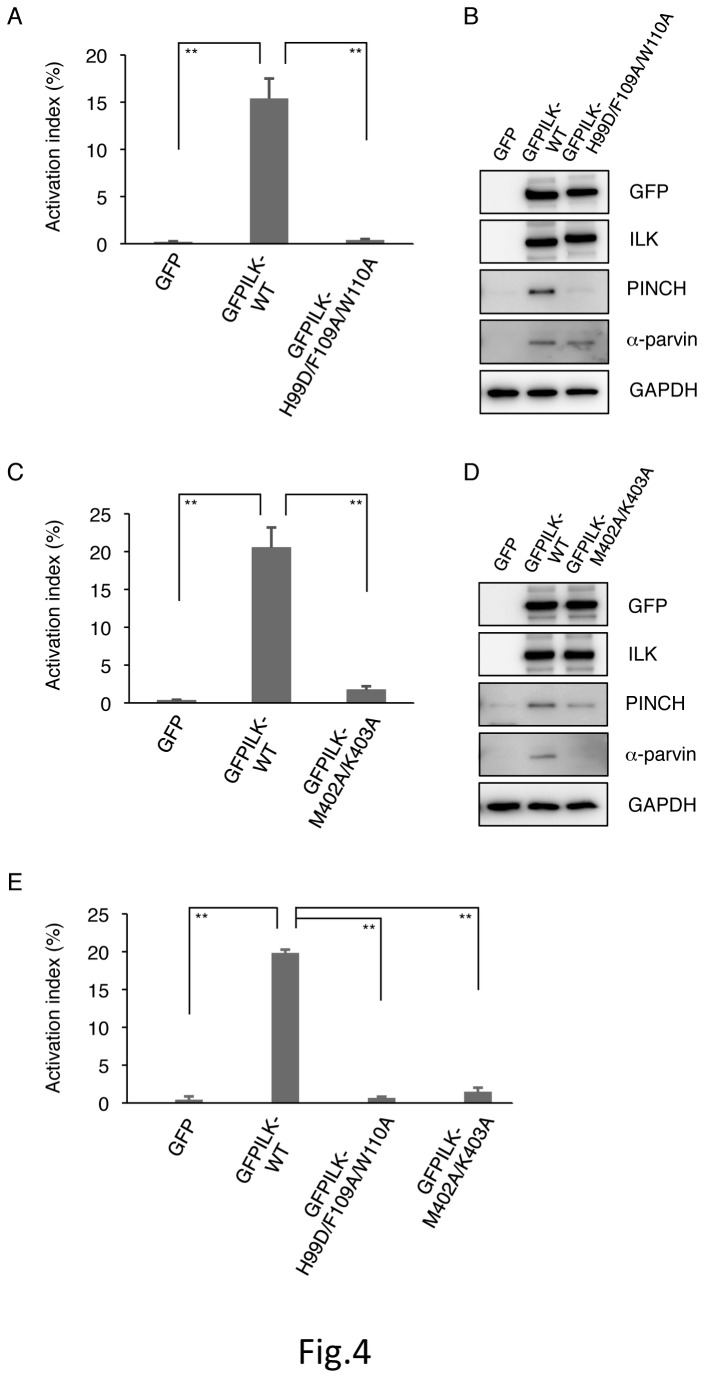
Effects of ILK mutants with defects in either PINCH or parvin binding. The activation indexes of transfected cells (A, C, E). ILK-deficient mutant cells were transiently transfected with GFP cDNA, GFP-fused wild-type ILK (GFPILK-WT) cDNA, GFP-fused ILK mutant with defective PINCH binding (GFPILK-H99D/F109A/W110A) cDNA (A, E), or GFP-fused ILK mutant with defective parvin binding (GFPILK-M402A/K403A) cDNA (C, E). After transfection, the binding of either PAC-1 (A, C) or fibrinogen (E) to the cells was analyzed by flow cytometry. The activation index was determined by the formula shown in Materials and Methods. A value of 100% represents the maximal binding of PAC-1 or fibrinogen to the cells treated with dithiothreitol. Data represent means ± SD of three independent experiments. ** indicates *P* < 0.01. Immunoblotting showing protein expression of GFP (B, D), GFP-fused wild-type ILK (GFPILK-WT) (B, D), GFP-fused ILK mutant with defective PINCH binding (GFPILK-H99D/F109A/W110A) (B), and GFP-fused ILK mutant with defective parvin binding (GFPILK-M402A/K403A) (D) in ILK-deficient mutant cells. Cell lysates were electrophoresed and immunoblotted with indicated Abs.

 To examine the significance of ILK-PINCH binding for integrin activation, we generated a GFP-fused ILK mutant (GFPILK-H99D/F109A/W110A) in which mutations were introduced into the binding sites for the LIM1 domain of PINCH in the ankyrin repeat domain of ILK. This ILK mutant is designed to disrupt ILK-PINCH binding but not ILK-α-parvin binding. When GFPILK-WT cDNA was transfected into mutant cells, PAC-1 binding was increased (Figure 4A). Transfection of the GFPILK-H99D/F109A/W110A cDNA into mutant cells failed to recover PAC-1 binding and did not induce an obvious upregulation of PINCH expression, whereas the ILK mutant protein was well expressed and α-parvin was similarly increased compared to the case with GFPILK-WT cDNA transfection, indicating the ILK-α-parvin complex (Figure 4B). These data suggest that ILK-PINCH binding is required for stable PINCH expression even in the presence of ILK, as well as for αIIbα6Bβ3 activation in the CHO cell system. When cell lysates of the mutant cells transfected with the GFPILK-H99D/F109A/W110A cDNA was subjected to immunoprecipitation with anti-α-parvin Ab, the ILK mutant was coprecipitated (data not shown). In addition, we generated a GFP-fused ILK mutant (GFPILK-M402A/K403A) that disrupts the parvin binding and that impairs the localization of ILK to focal adhesions as shown in the previous report [35]. Transfection of GFPILK-M402A/K403A cDNA into mutant cells showed strongly impaired PAC-1 binding and did not induce an overt upregulation of α-parvin expression (Figure 4C, D). These data suggest that ILK-parvin binding is necessary for stable parvin expression, as well as for αIIbα6Bβ3 activation in the CHO cell system. Moreover, when fibrinogen, a natural ligand for αIIbβ3, was used instead of PAC-1 in these experiments, similar results were obtained (Figure 4E). 

### Analysis of ILK in inactive αIIbβ3-expressing CHO cells

**Figure 5:**
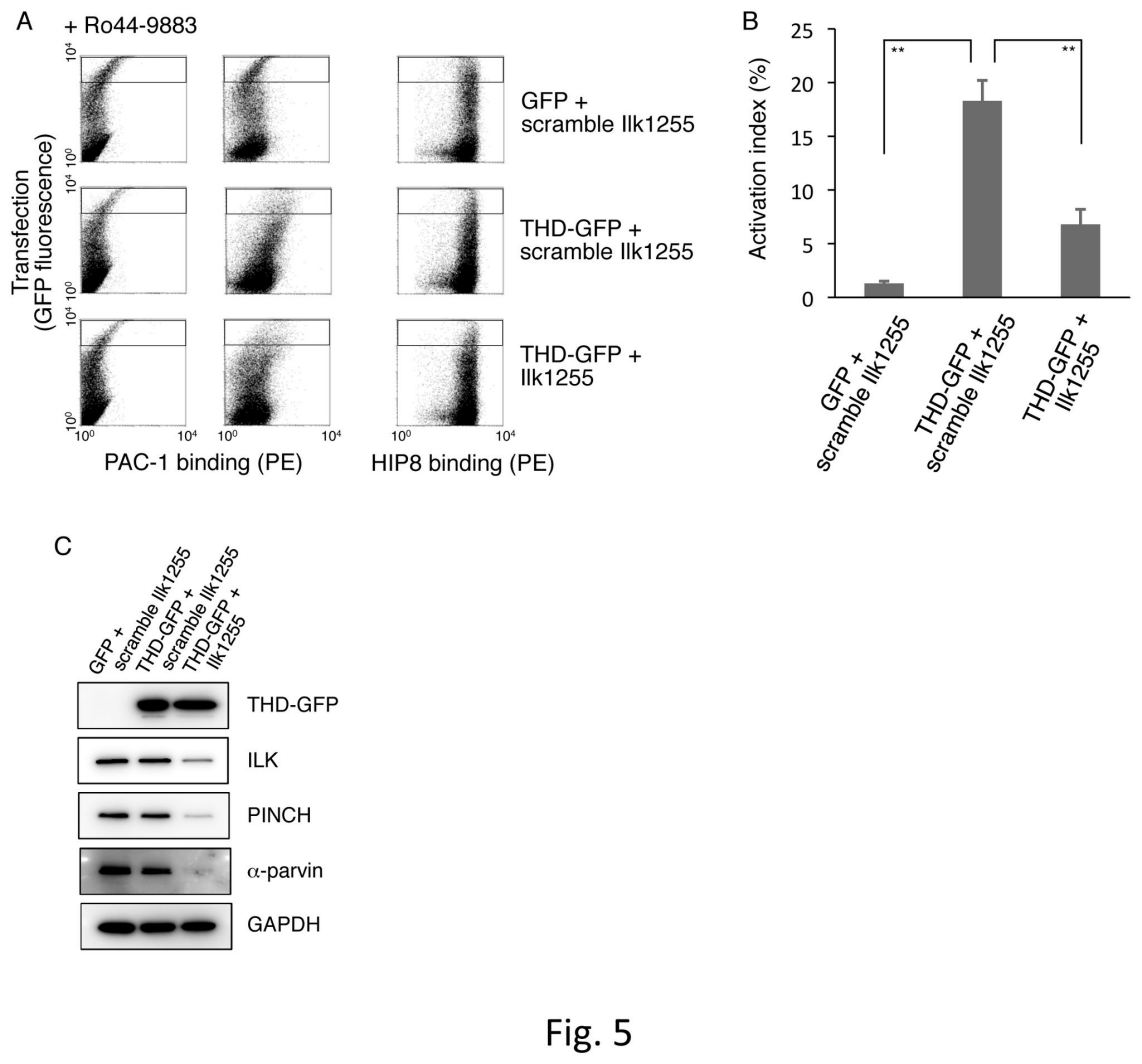
Knockdown effects of ILK on THD-mediated αIIbβ3 activation. (A) Dot plot detecting PAC-1 binding to cotransfected cells. Inactive αIIbβ3-expressing CHO cells were transiently cotransfected with GFP cDNA plus scrambled ILK siRNA (scramble Ilk1255), with THD-GFP cDNA plus scrambled ILK siRNA (scramble Ilk1255), or with THD-GFP cDNA plus ILK siRNA (Ilk1255). Highly transfected cells (cells in gated regions) were analyzed for PAC-1 binding or HIP8 (an αIIbβ3-specific mAb) binding. (B) The activation indexes of transfected cells. The activation index was determined by the formula shown in Materials and Methods. A value of 100% implies the median fluorescence intensity of HIP8 binding to the cells in gated regions. Data represent means ± SD of three independent experiments. ** indicates *P* < 0.01. (C) Immunoblotting to evaluate expression levels of IPP and THD-GFP. Cell lysates were electrophoresed and immunoblotted with indicated Abs.

 αIIbβ3 is present in an inactive state on CHO cells, and overexpression of the THD into those cells can induce αIIbβ3 activation [36]. The THD directly binds the integrin β3 cytoplasmic domain and causes integrin activation. To examine ILK’s contribution to THD-mediated αIIbβ3 activation, we performed knockdown experiments targeting ILK under transient THD expression (Figure 5). For the cotransfection of THD-GFP cDNA with scrambled ILK siRNA (scramble Ilk1255), the THD-GFP highly expressing cells exhibited a significant increase in the PAC-1 binding as compared to the case with GFP cDNA cotransfection (Figure 5A, B). In contrast, the cotransfection of THD-GFP cDNA with ILK siRNA (Ilk1255) showed decreased PAC-1 binding in the cells with high expression of THD-GFP (Figure 5A, B). The protein expression levels of ILK, PINCH, and α-parvin were suppressed by the cotransfection of ILK siRNA and THD-GFP cDNA, whereas the expression level of THD-GFP was not changed in the presence of ILK siRNA (Figure 5C). These results suggest that THD is sufficient to restore partially the integrin activation upon elimination of ILK by its siRNA (Ilk1255) and that ILK may assist THD in regulating the integrin activation state by assembling the IPP complex. In addition, these findings obtained from αIIbβ3-expressing CHO cells support the importance of the IPP complex observed in the αIIbα6Bβ3-expressing CHO cells. 　

### Overexpression of the IPP complex in inactive αIIbβ3-expressing CHO cells

**Figure 6:**
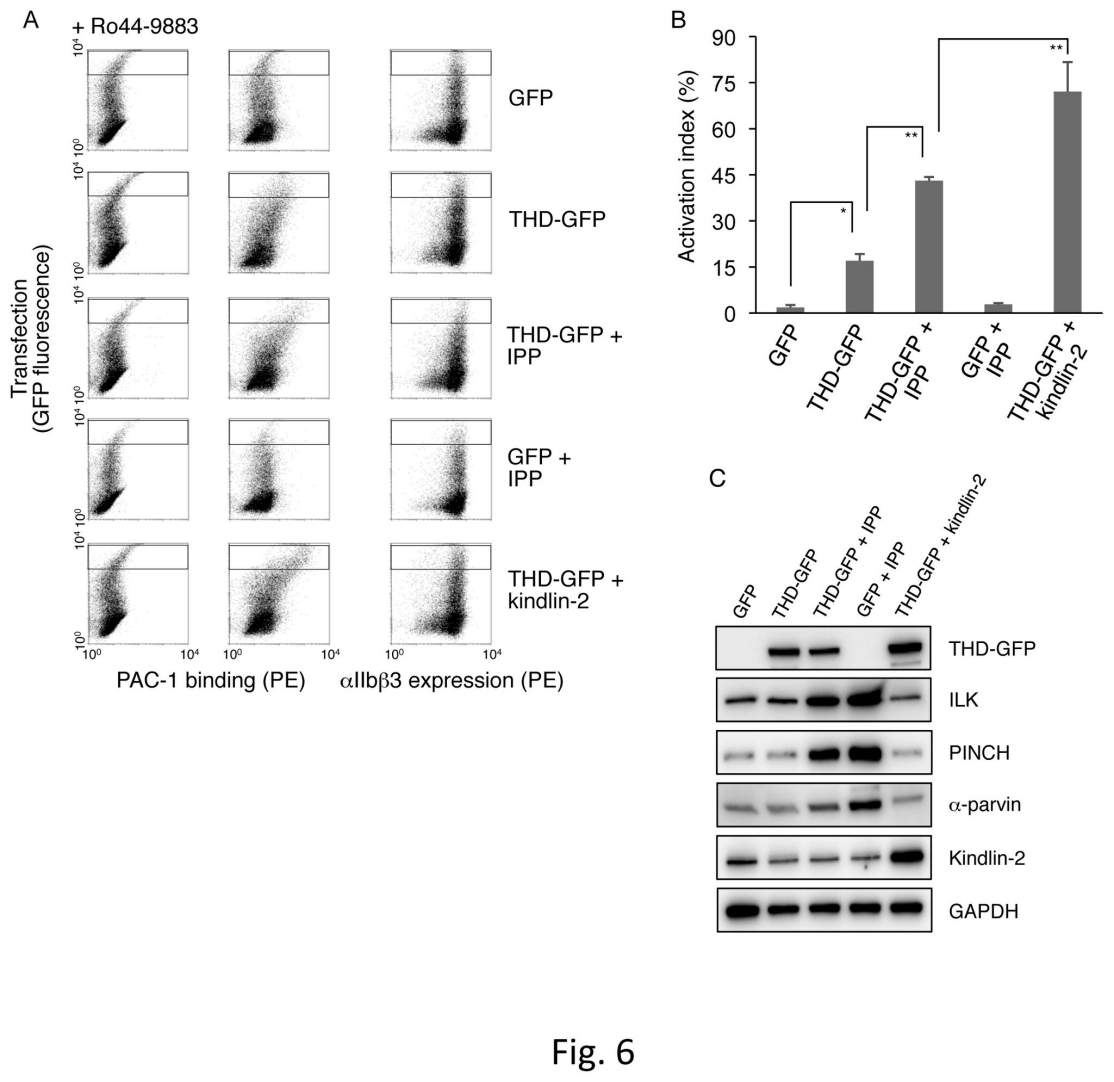
Effects of IPP overexpression on THD-mediated integrin activation in inactive αIIbβ3-expressing CHO cells. (A) Dot plot detecting PAC-1 binding to transfected cells. Inactive αIIbβ3-expressing CHO cells were transiently transfected with GFP cDNA, with THD-GFP cDNA, with THD-GFP cDNA plus IPP (ILK, PINCH, and α-parvin) cDNAs, with GFP cDNA plus IPP cDNAs, or with THD-GFP cDNA plus kindling-2 cDNA. Highly transfected cells in the gated regions were analyzed for PAC-1 binding or HIP8 (an αIIbβ3-specific mAb) binding. (B) The activation indexes of transfected cells. The index was determined by the formula shown in Materials and Methods. A value of 100% implies the median fluorescence intensity of HIP8 binding to the cells in gated regions. Data represent means ± SD of three independent experiments. * and ** indicate *P* < 0.05 and *P* < 0.01, respectively. (C) Immunoblotting to evaluate expression levels of IPP, THD-GFP, and kindlin-2. Cell lysates obtained from transfected cells were electrophoresed and immunoblotted with indicated Abs.

 To examine the IPP complex’s role in THD-mediated αIIbβ3 activation, we performed overexpression experiments of ILK, PINCH-1, and α-parvin in inactive αIIbβ3-expressing CHO cells. As expected, THD-GFP overexpression into αIIbβ3-expressing CHO cells induced PAC-1 binding in the cells with high expression of THD-GFP, as compared to the case with GFP cDNA transfection (Figure 6A, B). Interestingly, although quadruple overexpression of GFP and IPP did not significantly increase PAC-1 binding, quadruple overexpression of THD-GFP and IPP caused higher PAC-1 binding compared to the case with THD-GFP overexpression, suggesting a supportive effect of IPP on THD-mediated αIIbβ3 activation (Figure 6A, B). Kindlin-2 binds to the integrin β3 cytoplasmic domain and functions as a co-activator of talin [12,37]. As expected, double overexpression of THD-GFP and kindlin-2 cooperatively increased PAC-1 binding (Figure 6A, B), suggesting that both THD and kindlin-2 are required for the full activation of αIIbβ3. Regarding protein expression, THD-GFP was adequately expressed in each transfection, and the expression levels of ILK, PINCH, α-parvin, and kindlin-2 were higher than their endogenous expression levels in the cells with indicated transfection (Figure 6C). Thus, these data suggest that the IPP complex supports the THD for integrin αIIbβ3 activation. 

## Discussion

ILK is a multidomain scaffold protein that interacts with several cytoplasmic proteins [38,39]. In integrin adhesion sites, ILK exists in a ternary complex composed of the two other proteins PINCH and parvin. The ternary complex formation can stabilize each component and exert its function. In fact, ILK-deficient mutant CHO cells exhibited profoundly reduced PINCH and α-parvin expression levels, leading to inactive αIIbα6Bβ3 (Figure 1). The introduction of ILK expression into ILK-deficient mutant cells increased the expression levels of PINCH and α-parvin, accompanied by αIIbα6Bβ3 activation (Figure 1). The involvement of the IPP complex formation in integrin activation was confirmed in the knockdown experiments of PINCH and parvins in αIIbα6Bβ3-active parental cells (Figure 3). The ILK mutants with defects in either PINCH or parvin binding did not activate αIIbα6Bβ3 in ILK-deficient mutant cells (Figure 4). Since it has been reported that the parvin-binding defective ILK mutant (M402A/K403A) fails to localize to focal adhesions [35], these two ILK mutants are probably not recruited to the αIIbα6Bβ3 sites in a process of integrin activation. Thus, our data support a previous report that the proper complex formation of ILK, PINCH, and parvin is necessary for ILK recruitment to the integrin adhesion sites [35,40].

 Recent studies of integrin regulatory proteins have shown that both talin and kindlins directly bind to different regions in the integrin β cytoplasmic domain and cooperate in a final step of integrin activation [9,12]. In our experiments, kindlin-2 knockdown in αIIbα6Bβ3-active parental cells reduced the activation state of αIIbα6Bβ3, and talin knockdown exhibited similar results in our previous work [31]. The knockdown of PINCH and of parvins in the IPP complex decreased the activation state of αIIbα6Bβ3 in parental cells to a similar extent as did the knockdown of either kindlin-2 or talin. In inactive αIIbβ3-expressing CHO cells, overexpression of THD-GFP induced αIIbβ3 activation, and ILK knockdown reduced THD-GFP-mediated αIIbβ3 activation (Figure 5). Moreover, overexpression of the IPP complex further augmented the activation state of αIIbβ3 induced by the THD in inactive αIIbβ3-expressing CHO cells (Figure 6). These data suggest that the IPP complex participates in the cooperation of talin and kindlin-2 during the activation processes of not only αIIbα6Bβ3 but also αIIbβ3. The precise binding sites of ILK in the integrin β cytoplasmic domain remain to be determined, although their interaction has been reported [19]. There seem to be two possible direct and indirect manners of ILK binding to the integrin cytoplasmic domain. It was recently reported that the binding of PAT-4 (ILK) to UNC-112 (kindlin) in *C. elegans* is necessary for UNC-112 recruitment to adhesion sites [41]. While kindlin alone appears to bind to integrin in mammalian cells, the IPP complex would contribute to effective binding of kindlin to the β integrin cytoplasmic domain to fully induce conformational changes of integrin. 

 Adaptor proteins, PINCH-1 and -2, share high amino acid sequence identity [27]. Those are ubiquitously expressed in different tissues and show overlapping expression in many tissues. Both isoforms bind equally well to ILK, but its binding is mutually exclusive [34,42]. PINCH-1 is expressed in hematopoietic systems, and strong expression of it has been observed in megakaryocytes during fetal liver hematopoiesis [27]. PINCH-2 also joins in the IPP complex and contributes to the stabilization of individual proteins. We examined only PINCH-1 expression in CHO cells since we were unable to find PINCH-2 mRNA in parental CHO cells. Our knockdown experiment using PINCH-1-specific siRNA revealed the reduction of both ILK and α-parvin expression levels. In addition, published amino acid sequences of hamster PINCH-2 (GenBank accession number EGW10997) showed an amino acid length composed of 144 residues, shorter than that of mouse PINCH-2 (accession number NP659111) composed of 341 residues. This suggested that proper PINCH-2 may not be expressed in CHO cells. Unlike PINCH, α- and β-parvins were expressed in CHO cells and the knockdown of both parvins but not either α- or β-parvin decreased ILK and PINCH expression to a similar extent as the parvins. Thus, the parvins are complemented with each other in the formation of the IPP complex, and either one seems to support integrin activation by maintaining the IPP complex. 

 Platelets are likely to have α- and β-parvins, and both parvins contribute to the formation of the IPP complex [43,44]. The functional importance of the IPP complex for platelet integrin regulation has not been fully elucidated. There are only a few reports in which the IPP complex stably exists to a similar extent between resting and stimulated platelets [43,44]. It has been shown in human platelets that ILK is activated and binds to the β subunit of αIIbβ3 and the integrin collagen receptor α2β1 after stimulation with thrombin, phorbol 12-myristrate 13-acetate, and collagen [45,46]. These processes seem to aggregation-dependently occur in αIIbβ3 or aggregation-independently arise in α2β1. In a recent study using an ILK-conditional knockout mouse, ILK-deficient platelets exhibited reduced abilities of aggregation, fibrinogen binding, and α-granule secretion [33]. The ILK-deficient platelets also showed decreased expression levels of PINCH and α-parvin, suggesting that the IPP complex is involved in the regulation of integrin affinity. In platelets, the IPP complex may be translocated from the cytoplasm to the integrin β cytoplasmic domain in response to agonist stimulation and may participate in the control of integrin affinity. 

 The important role of ILK as a signal regulatory protein has been well investigated. Most works demonstrated that ILK functions in outside-in signaling through integrins [20,25,26,47-50]. In the present study, we showed that ILK supports integrin activation by assembling the IPP complex in the CHO cells. This effect may occur through a process in which kindlin-2 recruits ILK to integrins [37,51]. The IPP complex may participate in the cooperation of talin and kindlins in a final step of integrin activation and stabilize the active conformation of integrin. 
